# Test-Retest-Reliability of Video-Oculography During Free Visual Exploration in Right-Hemispheric Stroke Patients With Neglect

**DOI:** 10.3389/fnins.2020.00731

**Published:** 2020-07-21

**Authors:** Brigitte Charlotte Kaufmann, Dario Cazzoli, René Martin Müri, Tobias Nef, Thomas Nyffeler

**Affiliations:** ^1^Perception and Eye Movement Laboratory, Department of Neurology and BioMedical Research, University of Bern, Bern, Switzerland; ^2^Neurocenter, Luzerner Kantonsspital, Lucerne, Switzerland; ^3^Gerontechnology and Rehabilitation Group, ARTORG Center for Biomedical Engineering Research, University of Bern, Bern, Switzerland

**Keywords:** test-retest-reliability, video-oculography, mean gaze position, free visual exploration, neglect

## Abstract

The Mean gaze position during free visual exploration (FVE) is a sensitive tool to detect neglect in patients after a right-hemispheric stroke. Here we investigated the test-retest-reliability of mean gaze position during FVE in 23 patients with left-sided neglect after a first-ever sub-acute right-hemispheric stroke. We analyzed the reliability between different test sets administered within 11 days (test sets A and B, each including different images and their mirrored versions), and between repeated measures using the same test set administered three times within 2 days (test set C, including the same images and their mirrored versions). The intra-class correlation coefficient (ICC) showed good reliability between the two different test sets (test sets A and B; ICC = 0.819), and excellent reliability for the repeated measures with the same test set C (ICC = 0.964). FVE can therefore be recommended for the longitudinal assessments of patients’ neglect severity during neurorehabilitation as well as in treatment trials.

## Introduction

Spatial neglect is characterized by the failure to attend or respond to the contralesional hemispace (Heilman et al., 1993). After stroke, neglect has been reported to occur in 43–80% of patients with a right-hemispheric lesion (Stone et al., 1991; Azouvi et al., 2002; Ringman et al., 2004). Recent studies suggested that video-oculography may be an appropriate method to analyze visual neglect (visual exploration of naturalistic scenes, e.g., Delazer et al., 2018; Kaufmann et al., 2020a, b; visual exploration of abstract imags, e.g., Mannan et al., 2005; visual exploration of faces, e.g., Van Belle et al., 2010a, b; Verfaillie et al., 2014). Especially, a free visual exploration paradigm (FVE) might be a fast and accurate screening tool to detect neglect (Delazer et al., 2018; Kaufmann et al., 2020a). Indeed, evidence of the relationship between mean horizontal gaze position and neglect in everyday behavior, as assessed by means of the Catherine Bergego Scale (CBS), has been recently shown (Kaufmann et al., 2020a). Furthermore, the mean gaze position on the horizontal axis has been shown to be more sensitive in detecting neglect than conventional neuropsychological paper-pencil tests, such as the Line Bisection, Bells Cancellation, or Random Shape Cancellation Test (Kaufmann et al., 2020a). For most of these neuropsychological paper-pencil tests, test-retest-reliability is well known in neglect patients. However, the test-retest-reliability of the mean gaze position during FVE in neglect patients remains unknown. This aspect is of high relevance if the mean gaze position during FVE is intended to be used in clinical practice or as an outcome measure in research. Therefore, the aim of the present study was to investigate the test-retest-reliability of the mean gaze position on the horizontal axis as an indicator of neglect, as assessed by video-oculography during FVE. Test-retest-reliability was assessed between different test versions (i.e., including different images and their mirrored versions), as well as for the same test version applied over several measurement time points (i.e., including the same images and their mirrored versions) in patients with left-sided neglect after a first-ever subacute, right hemispheric stroke.

## Materials and Methods

### Patients

A total of 23 patients with left-sided neglect after a first-ever, right hemispheric, subacute stroke were recruited in the Neurorehabilitation Center of the Luzerner Kantonsspital (mean age = 72.74, SD = 10.25; 5 female; mean time since stroke = 19.73 days, SD = 8.83).

Neglect was diagnosed if patients showed a pathological score in at least one of the following tests: Catherine Bergego Scale (CBS > 1; Azouvi et al., 2003), Line Bisection Test (relative rightward deviation >11%; Wilson et al., 1987), Letter Cancellation Test (Center of Cancellation CoC > 0.083; Weintraub and Mesulam, 1985; Rorden and Karnath, 2010), Bells Cancellation Test (CoC > 0.081; Gauthier et al., 1989; Rorden and Karnath, 2010), or Random Shape Cancellation Test (CoC > 0.081; Weintraub and Mesulam, 1988). Individual test scores and patients’ characteristics are presented in Table 1. A further inclusion criterion was normal or corrected-to-normal visual acuity. Patients with a psychiatric disease were excluded. Visual field defects were assessed by means of Goldmann perimetry (isopter III/4) (four patients with incomplete hemianopia; five patients with quadrantanopia; see Table 1).

**TABLE 1:** Demographic and clinical data of neglect patients included in this study.

Patient Code	Age range	Sex	Handed-ness	Time since stroke (d)	Stroke type	Visual Field Defects	CBS	Line Bisection	Letter	Bells	Random Shape	Initial FVE
Pat_01	71–80	m	R	13	H	No	**16**	4.31	–0.01	0.00	0.01	0.96
Pat_02	61–70	m	R	27	I	Hemianopia	**14**	**53.18**	**0.69**	**0.35**	**0.71**	**1.91**
Pat_03	71–80	m	R	20	I	No	**26**	**63.65**	**0.77**	**0.87**	**0.75**	**8.05**
Pat_04	71–80	w	R	24	I	No	**12**	**45.24**	**0.89**	**−−**	**−−**	**6.36**
Pat_05	71–80	m	R	24	I	No	**18**	**14.21**	**0.15**	0.00	**0.19**	**8.27**
Pat_06	81–90	m	R	28	I	No	**16**	**49.04**	–0.03	0.07	–0.01	**2.11**
Pat_07	61–70	m	R	16	I	No	**3**	4.06	**0.58**	**0.16**	0.00	**4.38**
Pat_08	71–80	m	R	34	H	Inferior quadrantanopia	**26**	**18.93**	**0.23**	**0.72**	**0.38**	**5.71**
Pat_09	71–80	m	R	12	I	No	**4**	–2.69	0.03	0.04	0.03	**1.99**
Pat_10	71–80	w	R	13	I	No	**3**	**15.1**	0.03	**0.12**	0.04	**4.65**
Pat_11	81–90	m	R	37	H	No	**20**	**82.6**	**0.15**	**0.36**	**0.41**	**4.25**
Pat_12	71–80	m	R	20	I	Inferior quadrantanopia	**4**	7.01	0.05	**0.13**	–0.02	0.96
Pat_13	51–60	w	R	32	H	No	**16**	**94.82**	**0.46**	**0.15**	0.02	**3.49**
Pat_14	71–80	m	R	6	I	Hemianopia	**17**	**78.31**	**0.94**	**0.83**	**0.63**	–0.70
Pat_15	81–90	w	R	13	I	No	**3**	1.87	**0.09**	0.00	0.00	**2.31**
Pat_16	71–80	w	R	4	H	No	**19**	**20.21**	**0.69**	**0.79**	**0.85**	**2.04**
Pat_17	61–70	m	R	17	I	Hemianopia	**3**	3.65	0.06	–0.01	**0.17**	**1.81**
Pat_18	81–90	m	B	15	I	Hemianopia	**20**	–7.05	0.07	**0.13**	**0.66**	**2.22**
Pat_19	61–70	m	R	17	H	Inferior quadrantanopia (central 20° intact)	**11**	**16.58**	–0.06	**0.15**	0.05	**1.56**
Pat_20	51–60	m	R	17	H	Superior quadrantanopia	**19**	8.78	0.06	**0.31**	**0.28**	**2.05**
Pat_21	41–50	m	R	31	H	No	**20**	**19.25**	0.06	0.07	0.02	**2.98**
Pat_22	81–90	m	R	23	I	No	**18**	5.04	**0.75**	**0.56**	**0.86**	**4.31**
Pat_23	61–70	m	R	11	H	Inferior quadrantanopia	**5**	**12.27**	0.03	–0.01	–0.01	–0.31

**Mean (SD)**	**72.74 (10.25)**			**19.73 (8.83)**			**13.61 (7.61)**	**25.60 (30.13)**	**0.26 (0.32)**	**0.26 (0.30)**	**0.27 (0.32)**	**3.10 (2.36)**

*Table shows patients’ individual demographic data(age, sex, handedness), time since stroke, stroke type (H = hemorrhagic, I = ischemic), presence/absence of visual field defects assessed by means of Goldmann perimetry (isopter III/4), and their individual neglect severity score assessed with the Catherine Bergego Scale [CBS; range 0–30, neglect cut-off CBS ≥ 1 (Azouvi et al., 2003)], as well as their individual scores for the following neuropsychological paper-pencil tests: Line Bisection [neglect cut-off: relative rightward deviation of >11% (Wilson et al., 1987)], Letter Cancelation Test [neglect cut-off by means of Center of Cancellation (CoC): CoC > 0.083 (Weintraub and Mesulam, 1985)], Bells Test [neglect cut-off: CoC > 0.081 (Gauthier et al., 1989)], and Random Shape Cancelation test [neglect cut-off: CoC > 0.081 (Weintraub and Mesulam, 1988), and the mean gaze position (in degrees of visual angle) in the initial assessment of Free Visual Exploration neglect cut-off ≥1.333° (Kaufmann et al., 2020a)]. Individual test scores indicating neglect are displayed in bold typeface.*

All patients provided written informed consent to participate in the study. The study was approved by the local ethics committee.

### Video-Oculography

Video-oculography was assessed by means of an FVE paradigm, as previously described (Ptak et al., 2009; Cazzoli et al., 2011; Fellrath and Ptak, 2015; Paladini et al., 2019; Kaufmann et al., 2020a, b). In short, naturalistic images (e.g., colored photographs of everyday scenes such as the view of a mountain or of a public place; size 1200 × 900 pixels), and their mirrored versions (mirrored along the vertical axis) were presented on a computer screen. Each of the images was presented for 7 s, and was preceded by a central, black fixation-cross on a gray background (3 s), in order to enforce a common central starting point of visual exploration for all patients. All patients were instructed to freely explore the images, as if they would look at pictures in a newspaper or a photo album. A 3 × 3-point grid was presented for calibration of the eye-tracking system and for its validation prior to the experiment. During video-oculography, patients were seated in front of the screen, and their heads were positioned on a chin-and-forehead rest, to ensure that their mid-sagittal plane was aligned with the middle of the screen at a constant distance of 68 cm (resulting in a viewing angle of 28° × 21°) and to minimize head movements. Eye movements were recorded using a remote, infrared-based, video-eye-tracking system (EyeLink 1000 Plus System, SR Research, Ottawa, ON, Canada). All fixations with a duration between 100 and 2000 msec were included in the off-line data analyses (fixations excluded = 5.94%) (Salthouse and Ellis, 1980; Carpenter, 1988). The mean gaze position on the horizontal axis in degrees of visual angle (i.e., the mean x-position on the screen) was calculated using R. The mean gaze position, expressed in degrees of visual angle, allows quantifying neglect severity. The mean gaze position can range between −14° (at the far left of the images) to +14° (at the far right of the images). A mean gaze position of 0° thus indicates a spatially unbiased distribution of fixations, whereas positive values indicate a shift toward the right side of space, which is typical for right-hemispheric stroke patients with left-sided neglect (e.g., Paladini et al., 2019; Kaufmann et al., 2020a).

In a recent study, we also found a significant relationship between the mean gaze position and neglect severity in daily living as assessed by the CBS (Kaufmann et al., 2020a). Therefore, the Pearson’s correlation between the mean gaze position (in degrees of visual angle) of the initial assessment of FVE and the CBS total score was computed (1-tailed).

To investigate the test-retest-reliability of video-oculography during FVE between different test versions and over several measurement points, different test sets were used.

#### Test Sets A and B

**FIGURE 1:**
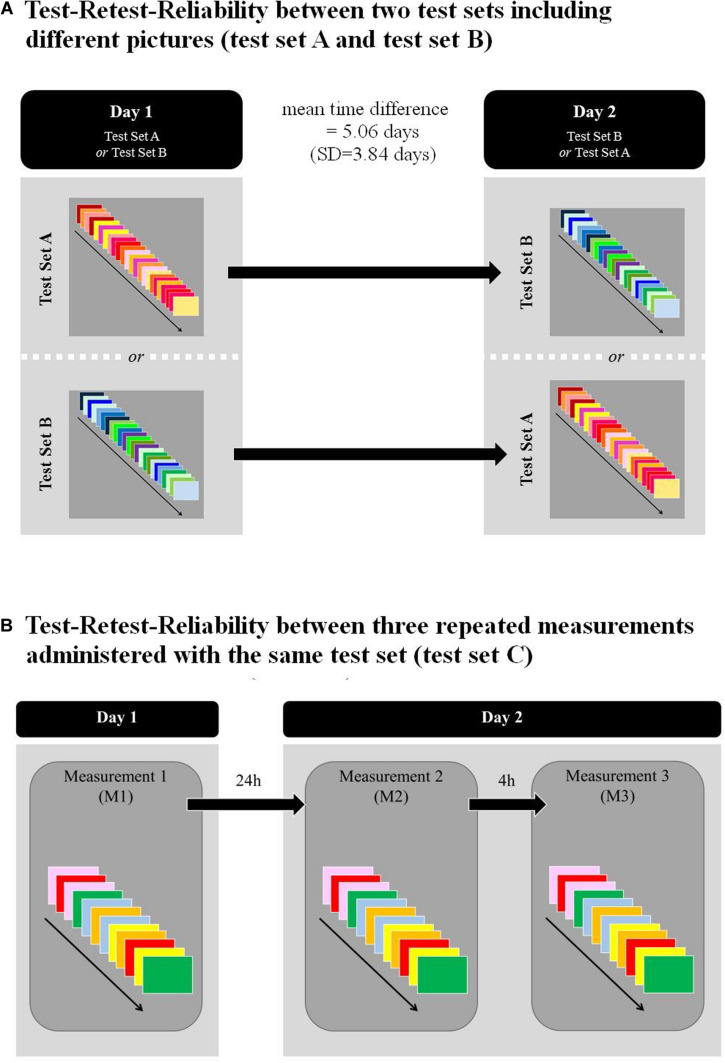
Shows the study designs for: **(A)** Test-retest-reliability between two different test sets (test sets A and test set B), calculated based on FVE administered in 2 days (mean time difference = 5.06, SD = 3.84). Patients were randomly assigned to one of the two test sets; **(B)** Test-retest-reliability between three measurement time points assessed with the same test set. All the measurements were administered in 2 consecutive days. Time between M1 and M2 was 24 h; time between M2 and M3 was 4 h.

In a first study, test performance in 18 patients was compared between two different test sets, i.e., test set A and test set B, using a cross-over design (Figure 1A). Each test set included 24 images (12 images and their 12 mirrored versions). All patients viewed both test sets within 11 days, the order of the sets being randomized over patients. The mean time elapsed between the two testing sessions with the respective test was 5.06 days (SD = 3.84 days).

#### Test Set C

In a second study, test-retest-reliability was assessed between three consecutive measurements, using test set C in 11 patients (thereof, six patients also participated in the first study). Test set C was a short version of FVE, including 12 images. Therefore, tests sets A and B were merged and six images and their six mirrored versions were randomly selected featuring test set C (Figure 1B). All three measurements were performed on 2 consecutive days in all patients. The mean time elapsed between M1 and M2 was thus 24 h, and between M2 and M3, 4 h, respectively (Figure 1B).

### Reliability Analysis

The mean gaze position (in degrees of visual angle) was compared between test sets A and B using a paired *t*-test (two-tailed). The mean gaze position (in degrees of visual angle) in the three measurements using test set C was evaluated by means of a univariate ANOVA with repeated measures. For all statistical tests, the significance level of α = 5% was used.

The reliability of video-oculography was determined by computing the intra-class correlation coefficient (ICC) using SPSS (IBM SPSS Statistics, Version 25) based on a mean-rating, absolute-agreement, two-way mixed-effects model (Koo and Li, 2016). Several reliability analyses were conducted. For each analysis, the patient’s individual agreements between measurements were plotted in a Bland-Altman plot including the 95% limit of agreement (Giavarina, 2015). The Bland-Altman plots allow comparing two measures of the same variable, by plotting the mean of the two measures on the *x*-axis and the difference between the two measures on the *y*-axis (Giavarina, 2015; Kalra, 2017). The graphic interpretation of the plots may then be used to identify outliers and potential, systematic over-/under-estimations in either of the two measures (Kalra, 2017).

#### Test-Retest-Reliability Between Two Test Sets Including Different Pictures (Test Set A and Test Set B)

The reliability of video-oculography between test set A and test set B was determined through the ICC. For each patient, the agreement between test set A and test set B was plotted in a Bland-Altman plot (Giavarina, 2015).

#### Test-Retest-Reliability Between Three Measurements Administered With the Same Test Set (C)

The reliability of video-oculography between three repeated measures of the same test set (C) was determined through the ICC. For each patient, the agreement between measurements was plotted for each combination (M1-M2, M1-M3, and M2-M3) separately using Bland-Altman plots (Giavarina, 2015).

## Results

The mean gaze position (in degrees of visual angle) in the initial assessment of FVE significantly correlates with neglect severity in daily living as assessed by the CBS (*r* = 0.362 moderate effect, *p* = 0.045, one-tailed, Table 1).

### Test-Retest-Reliability Between Two Test Sets Including Different Pictures (Test Set A and Test Set B)

The mean gaze position did not differ between test sets A and B [mean gaze position in test set A = 2.016° (SD = 1.458°), mean gaze position in set B = 2.134° (SD = 1.972°); *t*(17) = −0.370, *p* = 0.716]. Thus, in both sets, the spatial distribution of fixations is significantly shifted toward the right.

**FIGURE 2:**
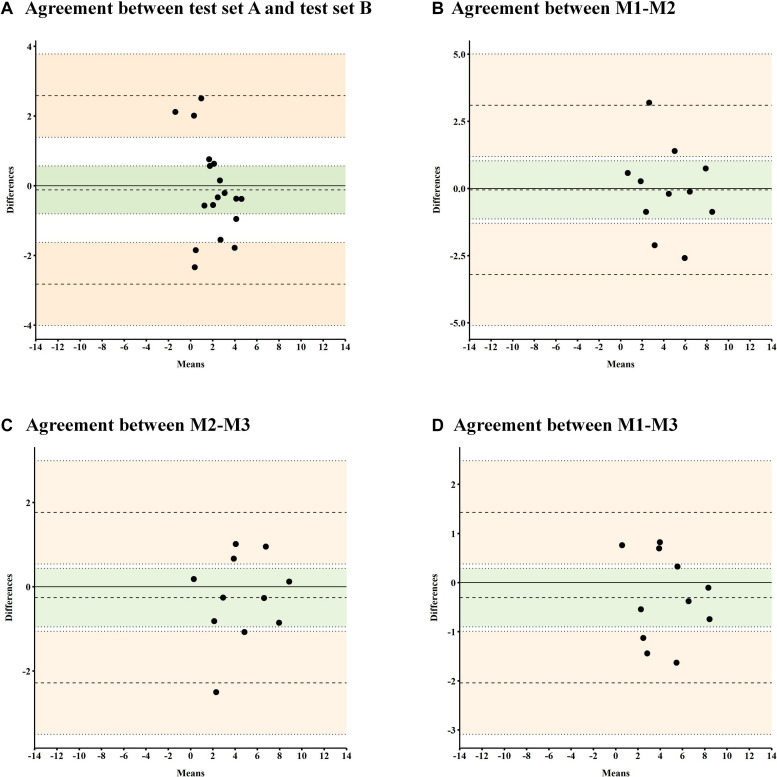
Bland-Altman plot for test-retest-reliability between two test sets including different pictures (test set A and test set B) and for test-retest-reliability between three measurements administered with the same test set (C). **(A)** Bland-Altman Plot showing the agreement between the mean gaze positions (in degrees of visual angle) assessed with test set A and test set B. **(B–D)** Bland-Altman Plot showing the agreement between the mean gaze positions (in degrees of visual angle) assessed in three repeated measures (M1, M2, M3) with the same test set (C). In the Bland-Altman plot, the difference of the paired measurements is plotted against the mean of these measurements. The bold line represents the 0 line, representing no difference between measures. The needled lines represent the mean of the difference ±1.96 SD (i.e., limits of agreement). The green area represents the mean differences and the upper/lower boundaries for the 95% confidence intervals. The pale orange area represents the upper and lower limits of agreement ±1.96 SD.

Intra-class correlation coefficient conducted between the two test sets of FVE (test sets A and B) showed a reliability index of 0.819, indicating good test-retest reliability for FVE (Koo and Li, 2016; Table 2). Ninety-five percent of our sample showed an ICC between 0.512 and 0.933 (Figure 2A). For each patient, the agreements between two measurements were plotted in a Bland-Altman plot including the 95% limit of agreement (Giavarina, 2015). The graphic interpretation of the Bland-Altman plot confirms that all patients performed within the upper and lower limits of agreement. The individual values of each participant are distributed above and below the 0 line, which suggests that there is no consistent bias of one test set versus the other (Kalra, 2017).

**TABLE 2:** Test-Retest-Reliability and absolute agreement for FVE in 18 neglect patients comparing test sets A and B, and for FVE in 11 neglect patients over three measurement time points (test set C).

Test	ICC (95% CI)	SEM	95% CI for test-retest agreement
Test-Retest-Reliability between two test sets including different pictures (test set A and test set B)	0.819 (0.512–0.933)	0.73°	1.43°
Test- Retest-Reliability between three measurement time points (M1,M2,M3), administering the same test set C	0.964 (0.903–0.990)	0.49°	0.96°

### Test-Retest-Reliability Between Three Measurements Administered With the Same Test Set (C)

The mean gaze position did not differ between measures of test set C [M1 = 4.423° (SD = 2.511°), M2 = 4.471° (SD = 2.822°), M3 = 4.758° (SD = 2.628°); *F*(1.272,12.723) = 0.403, *p* = 0.586, partial η^2^ = 0.039]. In all three measures, the spatial distribution of fixations is significantly shifted toward the right.

The analysis of FVE between three measurements with the same test set C revealed an ICC reliability index of 0.964, indicating excellent test-retest-reliability (Koo and Li, 2016; Table 2). Ninety-five percent of our sample showed an ICC between 0.903 and 0.990, referring to an excellent consistency. For each patient, the agreements between two measurements (M1-M2, M1-M3, M2-M3) were plotted in a Bland-Altman plot including the 95% limit of agreement (Giavarina, 2015; Figures 2B–D). The graphic interpretation of the Bland-Altman plots confirms that all patients performed within the upper and lower limits of agreement. In all three plots, the individual values of each participant are distributed above and below the 0 line, which suggests that there is no consistent bias of one measurement versus the others (Kalra, 2017).

## Discussion

In the present study, we investigated the test-retest-reliability of video-oculography during FVE between different test sets (test set A, test set B) and between repeated measures using the same test set (test set C).

We found that the mean gaze position on the horizontal axis during FVE of naturalistic images (e.g., photographs of everyday scenes such as the view of a mountain or public places) and their mirrored versions shows good to excellent reliability and is stable concerning retesting.

The reliability between the two test sets (test set A, test set B), administered within 11 days, was good (ICC = 0.819). This shows that the content of naturalistic photographs imaging everyday scenes seems not to be crucial, provided that each picture is presented with its respective mirrored version.

Furthermore, test-retest-reliability with repeated measures of the same test set (C), administered three times over 2 consecutive days, showed excellent test-retest-reliability (ICC = 0.964). These results suggest that mean gaze position during FVE shows comparable reliability with commonly used paper-pencil tests such as Star Cancellation Test (ICC = 0.89), Line Bisection (ICC = 0.47–0.97), Bells Cancellation Test (ICC = 0.84), and Random Shape Cancellation (ICC = 0.83) (Bailey et al., 2004; Machner et al., 2012).

Furthermore, comparing the ICC of our two analyses revealed that the reliability between test set A and test set B was slightly lower than the reliability for the repeated measures using test set C. This difference in ICC may have different causes. For example, since the mean time elapsed between measurements was 5 days, and all patients had sub-acute stroke, it is possible that neglect severity already improved in some patients due to spontaneous neglect recovery or strategies learned in neurorehabilitation therapy (Bailey et al., 2004). Note that, due to ethical reasons, all our patients received neurorehabilitative therapy in between the assessments of test set A and test set B; this might have influenced neglect recovery. On the other hand, as the same test set (C) was administered three times within a relatively short time period (within 2 days), the patients’ individual differences between test and retest measures might rather be related to variations in attentional level over time (Bailey et al., 2004).

Using video-oculography during FVE has several advantages. First, mean horizontal gaze position significantly correlates with neglect severity in daily living as assessed by the CBS (Kaufmann et al., 2020a), which was also replicated in the present study. Second, it has high sensitivity and specificity to diagnose neglect after a stroke, and it is even more sensitive than conventional neuropsychological cancellation tests (Kaufmann et al., 2020a). Third, FVE can be performed in less than 10 min and has the potential to be used as a fast and accurate screening tool that allows the initiation of comprehensive diagnostics and therapy from early on (Kaufmann et al., 2020a). Finally, visual exploration is spontaneous and requires only little effort from the patient.

A potential limitation of our study is that we included a relatively small sample size and did not include a healthy control group.

In conclusion, our results show good to excellent test-retest-reliability of FVE, and the ICC of FVE values which are comparable to commonly used paper-pencil tests. FVE can therefore be recommended for the longitudinal assessments of a patient’s neglect severity during neurorehabilitation as well as in treatment trials.

## Data Availability Statement

Individual participant data collected in this study will not be distributed openly to conform to the data privacy statements signed by our participants. However, specific aspects of the anonymized datasets and codes supporting the findings presented in this paper will be shared upon request to TNy, email: thomas.nyffeler@luks.ch.

## Ethics Statement

The studies involving human participants were reviewed and approved by the Ethics Committee Nordwest and Zentralschweiz (EKNZ), Switzerland. The patients/participants provided their written informed consent to participate in this study.

## Author Contributions

BK, TNy, and DC contributed to the conception and design of the study and wrote the first draft of the manuscript. BK organized the database and performed the statistical analyses. TNe and RM wrote sections of the manuscript. DC, TNy, TNe, and RM critically revised the work for important intellectual content. All authors contributed to the manuscript revision, and read and approved the submitted version.

## Conflict of Interest

The authors declare that the research was conducted in the absence of any commercial or financial relationships that could be construed as a potential conflict of interest.

## References

[B1] Azouvi P. Olivier S. de Montety G. Samuel C. Louis-Dreyfus A. Tesio L. (2003). Behavioral assessment of unilateral neglect: study of the psychometric properties of the Catherine Bergego Scale. *Arch. Phys. Med. Rehabil.* 84 51–57. 10.1053/apmr.2003.50062 12589620

[B2] Azouvi P. Samuel C. Louis-Dreyfus A. Bernati T. Bartolomeo P. Beis J. M. (2002). Sensitivity of clinical and behavioural tests of spatial neglect after right hemisphere stroke. *J. Neurol. Neurosurg. Psychiatry* 73 160–166. 10.1136/jnnp.73.2.160 12122175 PMC1737990

[B3] Bailey M. Riddoch M. Crome P. (2004). Test-retest stability of three tests for unilateral visual neglect in patients with stroke: star cancellation, line bisection, and the baking tray task. *Neuropsychol. Rehabil.* 14 403–419. 10.1080/09602010343000282

[B4] Carpenter R. H. (1988). *Movements of the Eyes*, 2nd Edn London: Pion Limited.

[B5] Cazzoli D. Nyffeler T. Hess C. W. Müri R. M. (2011). Vertical bias in neglect: a question of time? *Neuropsychologia* 49 2369–2374. 10.1016/j.neuropsychologia.2011.04.010 21530558

[B6] Delazer M. Sojer M. Ellmerer P. Boehme B. Benke T. (2018). Eye-tracking provides a sensitive measure of exploration deficits after acute right MCA stroke. *Front. Neurol.* 9:359. 10.3389/fneur.2018.00359 29942277 PMC6004522

[B7] Fellrath J. Ptak R. (2015). The role of visual saliency for the allocation of attention: evidence from spatial neglect and hemianopia. *Neuropsychologia* 73 70–81. 10.1016/j.neuropsychologia.2015.05.003 25956677

[B8] Gauthier L. Dehaut F. Joanette Y. (1989). The Bells test: a quantitative and qualitative test for visual neglect. *Int. J. Clin. Neuropsychol.* 11 49–54.

[B9] Giavarina D. (2015). Understanding Bland Altman analysis. *Lessons biostat.* 25 141–151. 10.11613/BM.2015.015 26110027 PMC4470095

[B10] Heilman K. M. Watson R. T. Valenstein E. (1993). *Neglect and Related Disorders.* New York, NY: Oxford University Press.

[B11] Kalra A. (2017). Decoding the bland-altman plot: basic review. *J. Pract. Cardiovasc. Sci.* 3 36–38. 10.4103/jpcs.jpcs_11_17

[B12] Kaufmann B. C. Cazzoli D. Pflugshaupt T. Bohlhalter S. Vanbellingen T. Müri R. M. (2020a). Eyetracking during free visual exploration detects neglect more reliably than paper-pencil tests. *Cortex* 129 223–235. 10.1016/j.cortex.2020.04.021 32512414

[B13] Kaufmann B. C. Knobel S. E. J. Nef T. Müri R. M. Cazzoli D. Nyffeler T. (2020b). Visual exploration area in neglect: a new analysis method for video-oculography data based on foveal vision. *Front. Neurosci.* 13:1412. 10.3389/fnins.2019.01412 32038129 PMC6987148

[B14] Koo T. Li M. (2016). A guideline of selecting and reporting intraclass correlation coefficients for reliability research. *J. Chiropract. Med.* 15 155–163. 10.1016/j.jcm.2016.02.012 27330520 PMC4913118

[B15] Machner B. Mah Y. Gorgoraptis N. Husain M. (2012). How reliable is repeatd testing for hemispatial neglect? Implications for clinical follow-up and treatment trials. *J. Neurol. Neurosurg. Psychiatry* 83 1032–1033. 22923514 10.1136/jnnp-2012-303296

[B16] Mannan S. Mort D. Hodgson T. Driver J. Kennard C. Husain M. (2005). Revisiting previously searched locations in visual neglect: role of right parietal and frontal lesions in misjudging old locations as new. *J. Cogn. Neurosci.* 17 340–354. 10.1162/0898929053124983 15811244

[B17] Paladini R. E. Wyss P. Kaufmann B. C. Urwyler P. Nef T. Cazzoli D. (2019). Re-fixation and perseveration patterns in neglect patients during free visual exploration. *Eur. J. Neurosci.* 49 1244–1252. 10.1111/ejn.14309 30561071

[B18] Ptak R. Golay L. Müri R. M. Schnider A. (2009). Looking left with left neglect: the role of spatial attention when active vision selects local image features for fixation. *Cortex* 45 1156–1166. 10.1016/j.cortex.2008.10.001 19038381

[B19] Ringman J. M. Saver J. L. Woolson R. F. Clarke W. R. Adams H. P. (2004). Frequency, risk factors, anatomy, and course of unilateral neglect in an acute stroke cohort. *Neurology* 63 468–474. 10.1212/01.WNL.0000133011.10689.CE 15304577

[B20] Rorden C. Karnath H. O. (2010). A simple measure of neglect severity. *Neuropsychologia* 48 2758–2763. 10.1016/j.neuropsychologia.2010.04.018 20433859 PMC3129646

[B21] Salthouse T. A. Ellis C. L. (1980). Determinants of eye-fixation duration. *Am. J. Psychol.* 93 207–234. 7406068

[B22] Stone S. P. Wilson B. Wroot A. Halligan P. W. Lange L. S. Marshall J. C. (1991). The assessment of visuo-spatial neglect after acute stroke. *JNNP* 54 345–350. 10.1136/jnnp.54.4.345 2056321 PMC488491

[B23] Van Belle G. De Graef P. Verfaillie K. Busigny T. Rossion B. (2010a). Whole not hole: expert face recognition requires holistic perception. *Neuropsychologia* 48 2620–2629. 10.1016/j.neuropsychologia.2010.04.034 20457169

[B24] Van Belle G. De Graef P. Verfaillie K. Rossion B. Lefèvre P. (2010b). Face inversion impairs holistic percep-tion: evidence from gaze-contingent stimulation. *J. Vis.* 10 1–13. 10.1167/10.5.10 20616142

[B25] Verfaillie K. Huysegems S. De Graef P. Van Belle G. (2014). Impaired holistic and analytic face processing in congenital prosopagnosia: evidence from the eye-contingent mask/window paradigm. *Vis. Cogn.* 22 503–521. 10.1080/13506285.2014.881446

[B26] Weintraub S. Mesulam M. (1985). *Mental State Assessment of Young and Elderly Adults in Behavioral Neurology* (I. M.-M. Mesulam Ed.). Philadelphia: F. A. Davis.

[B27] Weintraub S. Mesulam M. (1988). Visual hemispatial inattention: stimulus parameters and exploratory strategies. *J. Neurol. Neurosurg. Psychiatry* 51 1481–1488. 10.1136/jnnp.51.12.1481 3221214 PMC1032760

[B28] Wilson B. A. Cockburn J. Halligan P. W. (1987). Development of a behavioral test of visuospatial neglect. *Arch. Phys. Med. Rehabil.* 68 98–102. 3813864

